# Estrategia combinada para cesación tabáquica en un centro de atención primaria de Cataluña. Estudio piloto longitudinal retrospectivo

**DOI:** 10.1016/j.aprim.2025.103385

**Published:** 2026-01-13

**Authors:** Esther Sotillo Saez

**Affiliations:** Centre d’Atenció Primària (CAP) Tàrraco, Institut Català de la Salud (ICS), Tarragona, España

Según la Organización Mundial de la Salud (OMS), el consumo de tabaco es responsable de más de 8 millones de muertes anuales^1^. En 2024, un 28,6% de los hombres y un 20% de las mujeres ≥ 15 años eran fumadores en Cataluña^2^.

La atención primaria (AP) es el entorno idóneo para implementar estrategias de cesación tabáquica por su accesibilidad y capacidad de seguimiento longitudinal^1^. La incorporación de nuevos perfiles, como el de profesionales de Psicología General Sanitaria con función de Referente de Bienestar Emocional (RBEC), ha permitido desarrollar un modelo de atención sanitaria integral^3^. Este innovador enfoque combina el seguimiento individualizado realizado por enfermería con terapias grupales conducidas por RBEC, potenciando la percepción de autoeficacia de los pacientes, factor clave para mantener la motivación durante las etapas de cambio según el modelo transteórico de Prochaska y Di Clemente^4^.

La OMS, en sus primeras directrices clínicas publicadas en 2024 sobre cesación tabáquica, subraya que la combinación de tratamiento farmacológico e intervenciones conductuales incrementa significativamente las tasas de éxito en el abandono tabáquico^5^. En línea con estas recomendaciones, este estudio piloto pretendió evaluar si dicha combinación mejoraba los resultados de deshabituación.

Se llevó a cabo un estudio longitudinal retrospectivo en un centro de AP urbano de Cataluña. El reclutamiento de hizo desde junio de 2022 hasta marzo de 2023, incluyendo inicialmente a 98 pacientes fumadores (≥ 16 años) que acudieron a su centro de AP para dejar de fumar. Los participantes firmaron el consentimiento informado, que contó con la aprobación del comité ético (CEIm 24/295-P). Tras pérdidas durante seguimiento, se incluyó a 62 participantes (59,7% mujeres con edad 53,4 ± 11,7 años y 40,3% hombres con edad 48,2 ± 15,9 años).

La variable principal fue la abstinencia tabáquica. Las variables secundarias fueron: sexo, edad, grado de dependencia nicotínica (test de Fagerström), motivación (test de Richmond), intentos previos de abandono y tipo de estrategia terapéutica (farmacológica, conductual, combinación de ambas, o ninguna intervención) (material adicional, figura 1).

La recogida de datos combinó registros electrónicos del sistema eCAP (para información demográfica, clínica y de tratamiento) y entrevistas telefónicas semiestructuradas a los 12 y 24 meses de seguimiento (para estado de abstinencia tabáquica e intentos previos de cesación).

Los análisis descriptivos bivariados y de regresión estadística fueron ejecutados mediante el programa estadístico Stata v17.

Los análisis bivariados identificaron asociaciones estadísticamente significativas entre la motivación elevada pretratamiento y la abstinencia tabáquica, tanto a 12 meses como a 24 meses (p < 0,001) y entre el reclutamiento durante la Semana Mundial sin Tabaco y la abstinencia a los 12 meses (p < 0,05).

El modelo de regresión logística mostró asociación estadísticamente significativa (p < 0,05) entre la motivación para dejar de fumar y la abstinencia tabáquica sostenida en ambos periodos de seguimiento:

(OR = 3,1; IC 95%: 1,4-6,8) a 12 meses.
(OR = 3,3; IC 95%: 1,5-7,5) a 24 meses.

Nuestros datos no alcanzaron significación estadística para respaldar la superioridad del enfoque combinado —objetivo central de la investigación—, posiblemente debido a limitaciones en el tamaño muestral (n = 62), recolección incompleta de algunas pruebas en los participantes y realización en un único centro.

Los resultados confirman que la motivación constituye un factor determinante para lograr la abstinencia mantenida, lo que apoya el modelo transteórico^4^ y concuerdan con la literatura científica reciente^6^.

En futura investigación se ampliará el tamaño muestral hasta diciembre de 2027 (CEIm 24/295-P), se implementarán protocolos que aseguren la recogida de datos de adicción y motivación en todos los participantes y se aumentará el estudio a otros centros del área asistencial. De esta manera se podrán obtener resultados robustos y mayor validez externa para optimizar programas de deshabituación tabáquica en AP.

Estudio que deriva del proyecto «*Abordatge Integral i multidisciplinari de l’hàbit tabàquic en el CAP Tàrraco*» aprobado por el Comité de Ética del IDIAP Jordi Gol con Código CEIm:24/295-P.

## Financiación

Este estudio se desarrolló gracias al reconocimiento obtenido en la convocatoria 2022 para la promoción de proyectos de salud, impulsada desde la Estrategia de Salud Comunitaria a l’Atenció Primària i Comunitària (APIC). La financiación provino del proyecto premiado: «Abordatge integral i multidisciplinari de l’hàbit tabàquic en el CAP Tàrraco».

## Consideraciones éticas

Este estudio se ha realizado tras la aprobación del Comité Ético de Investigación Clínica del IDIAP JGol (CEIm) bajo código 24/295-P, garantizando el cumplimiento de los principios éticos fundamentales. La participación fue estrictamente voluntaria, requiriendo consentimiento informado escrito que detallaba objetivos, procedimientos, beneficios potenciales y riesgos asociados. La confidencialidad se aseguró mediante asignación de códigos numéricos únicos y acceso restringido exclusivamente al personal investigador autorizado, preservándose, en todo caso, el derecho de las personas participantes a retirarse en cualquier momento sin afectar su atención sanitaria.

La investigación cumple con la Declaración de Helsinki y la normativa de protección de datos (Ley Orgánica 3/2018). El documento de consentimiento cumple las buenas prácticas clínicas, incluyendo objetivos; intervenciones y seguimientos requeridos; beneficios individuales/colectivos; riesgos o molestias derivadas; derechos de acceso a datos; y medidas técnicas/organizativas implementadas para garantizar la seguridad de la información personal.

## Conflicto de intereses

No hay conflicto de intereses.
