# Supplementary material for: Combined strategy for smoking cessation in a Catalan primary care center. Retrospective longitudinal pilot study
Source: Aten Primaria. 2026 Jan 13;58(2):103385. [Article in Spanish] doi: 10.1016/j.aprim.2025.103385 (PMC12828736; doi:10.1016/j.aprim.2025.103385)
Supplement: Supplementary file 1 [file mmc1.doc]

**ANEXO**

**Figura 1.** *Esquema del estudio.*

Intervención

conductual

(n=20)

Combinación farmacológico y conductual (n=27)

Sin intervención (n=5)

Se incluyen en el estudio (n= 62)

Tipo de estrategia utilizada

No se pudo contactar con ellas n=22

Exitus n=4

No estaban asignadas al EAP n=9

No se incluyen en el estudio (n=35)

Tratamiento farmacológico (n=10)

Seguimiento a los 24 meses (n=62)

Seguimiento a los 12 meses (n=62)

Aceptan participar en el estudio (n= 97)

Personas fumadoras ≥ 16 años que se interesaron por los

tratamientos e intervenciones para dejar de fumar (n=108)

**PIE DE FIGURA**

**Figura 1.**

*Estudio longitudinal retrospectivo de mediciones repetidas de uno y dos años de seguimiento de personas fumadoras mayores de 16 años que obtuvieron tratamiento o intervención en un equipo de Atención Primaria urbano de Cataluña.*
